# Mediating Effect of Chronic Illnesses in the Relationship Between Psychological Distress and COVID-19 Vaccine Acceptance

**DOI:** 10.1177/10105395211047868

**Published:** 2021-09-22

**Authors:** Won Sun Chen, Ching Sin Siau, Suzanna Awang Bono, Wah Yun Low

**Affiliations:** 1 Swinburne University of Technology, Hawthorn, Victoria, Australia; 2 Universiti Kebangsaan Malaysia, Kuala Lumpur, Malaysia; 3 Universiti Sains Malaysia, Penang, Malaysia; 4 Asia-Europe Institute, Universiti Malaya, Kuala Lumpur, Malaysia; 5 Faculty of Medicine, Universiti Malaya, Kuala Lumpur, Malaysia

**Keywords:** COVID-19 vaccine acceptance, psychological distress, chronic illnesses, mediating effect, Malaysia

## Abstract

The impact of the COVID-19 pandemic on mental health is an emerging problem globally. This study aimed to examine the mediating effect of chronic illnesses in the relationship between psychological health and the acceptance of the COVID-19 vaccine, prior to the national vaccine rollout in Malaysia. An online cross-sectional study was conducted in Malaysia between December 10, 2020, and February 9, 2021. In addition to the descriptive analyses, a mediation analysis was performed to examine the mediating effect of chronic illnesses in the relationship between psychological distress and the willingness to accept the vaccine. A total of 1738 participants completed the survey. The psychological distress levels were found to be significantly different across demographic factors such as age, gender, and social economic status. This study demonstrated a partial mediating effect of chronic illnesses in the relationship between psychological distress and vaccine acceptance.

## What We Already Know

- People with chronic illnesses have been disproportionately affected by the COVID-19 pandemic. There are contrasting findings in the existing literature about how this vulnerable population reacts toward accepting the COVID-19 vaccine.
- Previous studies have highlighted the positive association between the intolerance of the uncertainty of the pandemic and psychological distress.
- Globally, countries are racing to increase the vaccination rate in their respective countries in order to achieve herd immunity. As of August 6, 2021, the COVID-19 vaccination rate in Malaysia remained relatively low at 25.6%.

## What This Article Adds

- This study highlighted about 14.4% of participants reported moderate to severe symptoms. This prevalence rate was comparable with the rate reported in Germany and Austria (19%), America (11.2%), and Saudi Arabia (14.5%), but a much lower rate was recorded in China (3.3%).
- This study suggested that chronic illnesses partly mediate the relationship between psychological distress and the willingness to accept the COVID-19 vaccine.
- In order to promote vaccine acceptance, more compelling content especially in relation to chronic illnesses should be included in the targeted health campaigns. With accurate information being disseminated, these campaigns are likely to reduce psychological distress and subsequently increase positive attitudes toward the COVID-19 vaccine.

## Introduction

The World Health Organization (WHO) has declared the coronavirus disease 2019 (COVID-19) as a global pandemic since March 2020.^1^ As of August 6, 2021, there were over 201 124 million confirmed COVID-19 cases worldwide with reported deaths of over 4.27 million.^2^ In Southeast Asia, countries that are reported more than 20 000 new cases per day as of August 6, 2021, included Indonesia (39 532 new cases per day), Thailand (21 379 new cases per day), and Malaysia (20 889 new cases per day). Additionally, the COVID-19 mortality rate for the top three countries are Indonesia (387.78 deaths per million population), Malaysia (321.12 deaths per million population), and the Philippines (269.12 deaths per million population). Unfortunately, the percentage of fully vaccinated adults in these countries remains relatively low: Malaysia (25.6%), Indonesia (8.3%), Thailand (6.0%), and the Philippines (9.8%).^2^ In Malaysia, there were 1 224 595 confirmed COVID-19 cases with over 10 000 deaths reported as of August 6, 2021. Since July 13, 2021, the number of COVID-19 cases has soared to a record high of more than 10 000 new cases per day.^3^

There is a global consensus that the COVID-19 vaccination is a crucial step forward to transition out of the pandemic. At the beginning of 2021, many countries have granted expedited approval of numerous vaccines to support the vaccine rollout.^4^ The success of a safe and efficacious COVID-19 vaccine rollout not only depends on the vaccines’ production and availability, but also the vaccine uptake. Although the WHO supports achieving herd immunity through COVID-19 vaccination, the percentage of people who need to be immunized against COVID-19 in order to achieve herd immunity remains unknown.^5^

The impact of the COVID-19 pandemic on mental health is an emerging problem globally. A recent Canadian study showed that there were clinically significant levels of anxiety across various age groups, 36% for those aged 15 to 34 years, 27% of people aged 35 to -54 years, and 15% of those 55 years and older. Younger people were reported to have more COVID-19-related worries than the older groups.^6^ Apart from focusing on the development of the vaccine to mitigate the physical health impacts of the pandemic, it is equally important to address the mental health impacts. A range of psychological health conditions are found to be associated with behavior and intentions toward the vaccine. For example, previous studies revealed the relationship between elevated psychological health and vaccine uptake intentions among health care workers^7^ and the general population.^8^ Furthermore, deteriorating psychological health due to the COVID-19 pandemic was found to be associated with reduced vaccine intake intention.^9,10^

Chronic illnesses are long-lasting conditions that could lead to persistent effects. Both social and economic consequences due to chronic illnesses can affect an individual’s overall quality of life. Studies in France and the Middle East found that individuals with chronic illnesses were less likely to accept the COVID-19 vaccine, but they were more likely to take personal health-protective measures against COVID-19.^11,12^ However, studies conducted in the United States showed a higher prevalence of vaccine acceptance among individuals with underlying chronic illnesses.^13,14^ In terms of gender, a previous study showed that the vaccine uptake was more likely among women in France, Germany, Russia, and Sweden in comparison to men. Furthermore, females with underlying chronic illnesses were found to have reduced odds of vaccine acceptance.^15^ On the other hand, highly educated individuals in Ecuador, France, Germany, India, and the United States were more likely to accept the vaccine.^16^

The effect of the COVID-19 pandemic on psychological health is likely to last for a longer period of time. Furthermore, it is equally important to examine the influence of psychological health toward vaccine uptake. Additional factors such as underlying chronic illnesses might play a crucial role in influencing vaccine uptake intentions. Therefore, this study aimed to examine the mediating effect of chronic illnesses in the relationship between psychological health and the acceptance of the vaccine, prior to the national rollout of the vaccination program in Malaysia.

## Methods

### Participants and Procedure

In order to cope with the massive COVID-19 outbreaks, the WHO has strongly recommended countries to implement measures such as lockdowns, stringent personal hand hygiene, and social distancing. It remains unclear to what degree countries adhere to those recommendations in reducing COVID-19 transmission. The International Citizen Project COVID-19 (ICPCovid; https://www.icpcovid.com/en/form/covid-19-vaccine-survey) is an international research consortium, which developed a series of large-scale online surveys to understand country-level adherence to the preventive measures recommended by the WHO. This particular survey consists of questions in relation to sociodemographic factors, COVID-19 perceptions, willingness of the population to be vaccinated, and reasons for vaccine hesitancy. Collaborating institutions are located in Brazil, Cameroon, Ecuador, Peru, Mali, Ghana, the Gambia, the Democratic Republic of Congo, Uganda, Burundi, Zambia, Malawi, Mozambique, South Africa, India, Thailand, Taiwan, Vietnam, Benin, Belgium, Somalia, Tunisia, Malaysia, and Bangladesh. To date, there are more than 20 publications using ICPCovid data.

This cross-sectional study was conducted as part of the Wave 1 of the ICPCovid surveys. Adult Malaysians, aged 18 years and older, were invited to complete the online questionnaire during the period of December 10, 2020, to February 9, 2021, through different social media platforms, such as WhatsApp, Facebook, SMS, Messenger, Twitter, Instagram, and university webpage portals. Those who consented were asked to complete the questionnaire described below.

### Measures

Part one of the questionnaire comprised demographic questions in relation to age, gender, chronic illnesses, highest education level, and socioeconomic status.

The second part of the questionnaire consisted of the Patient Health Questionnaire (PHQ-4), which is designed to assess psychological distress. This scale contains two items in relation to depression symptoms and another two items to assess anxiety symptoms.^17^ Each item was assessed using a 4-point Likert-type scale (0 = not at all, 3 = nearly every day). The total PHQ-4 score, tallied over the 4 items, yield a score ranging from 0 to 12. Broadly, psychological distress is categorized as normal (0-2), mild (3-5), moderate (6-8), and severe (9-12).^18^

The outcome measure of this study relied on a question pertaining to the willingness of participants to accept the COVID-19 vaccine with at least 95% effectiveness level.

### Statistical Analysis

Continuous variables were described by means, standard deviation, median, and range, while categorical variables were presented using frequency and percentage. Distributed differences of continuous variables were assessed using the Mann-Whitney *U* test or the Kruskal-Wallis test, and the χ^2^ test was performed for categorical variables.

The associations between psychological distress and the willingness to accept the vaccine was assessed visually using a clustered bar chart separately for participants with and without chronic illnesses. A χ^2^ test was used to quantify these associations.

The mediation analysis was conducted to examine the mediating effect of chronic illnesses in the relationship between psychological distress and the willingness to accept the vaccine.^19^ In this analysis, chronic illnesses, a binary variable recorded as “Yes” or “No,” was regarded as the mediating factor; the independent variable was psychological distress (PHQ-4 score) and willingness to accept COVID-19 vaccine with at least 95% effectiveness level was the outcome variable. Specifically, this mediation analysis consisted of four sets of logistic regression models: (1) to regress psychological distress to the willingness to accept the vaccine; (2) to regress psychological distress to the chronic illnesses; (3) to regress chronic illnesses to the willingness to accept the vaccine; and (4) to regress both psychological distress and the chronic illnesses to the willingness to accept the vaccine. Two subsequent models were constructed according to the following order: unadjusted (Model 1) and adjusted for significant demographic factors such as age, gender, and social economic status (Model 2). Odds ratios (ORs) and 95% confidence intervals (CIs) were presented for both models.

Diagnostic assessments for outliers, influential points, and multicollinearity were performed to validate the resulting models. A *P* value of less than .05 was deemed statistically significant for all 2-tailed tests. The analysis was conducted using IBM SPSS Statistics version 27 (IBM Corp).

## Results

### Descriptive Statistics

A total of 1738 participants completed the online survey. Table 1 shows that the average age of the participants was 41.1 years (SD ± 15.8 years); most were female (65.6%), slightly over 80% with tertiary education, and almost a quarter reported having at least one chronic illness. Furthermore, the summary of the demographic data suggested that younger participants reported having significantly higher levels of psychological distress (*H*[3] = 159.13, *P* <.001), with a significant difference also observed for gender (χ^2^[3] = 19.61, *P* < .001) and socioeconomic status (χ^2^[9] = 47.84, *P* < .001). However, the breakdown of the highest education level and chronic illnesses was found to be similar at varying levels of psychological distress.

**Table 1.** Demographic Characteristics and Psychological Distress of Participants.

Variables		Psychological distress				*P*
	Total (n = 1738)	Normal (n = 844)	Mild (n = 644)	Moderate (n = 175)	Severe (n = 75)	
Age, years, n (%)						
Mean ± SD	41.1 ± 15.8	45.9 ± 16.4	37.1 ± 13.5	36.2 ± 14.3	31.7 ± 14.1	
Median (min-max)	38 (18-87)	47 (18-87)	35 (18-75)	32 (18-73)	25 (19-72)	<.001^a^
18-29	540 (31.1%)	188 (22.3%)	231 (35.9%)	76 (43.4%)	45 (60.0%)	<.001^b^
30-39	378 (21.7%)	152 (18.0%)	170 (26.4%)	42 (24.0%)	14 (18.7%)	
40-49	243 (14.0%)	112 (13.3%)	112 (17.4%)	15 (8.6%)	4 (5.3%)	
50-59	259 (14.9%)	152 (18.0%)	74 (11.5%)	27 (15.4%)	6 (8.0%)	
≥60	318 (18.3%)	240 (28.4%)	47 (8.9%)	15 (8.6%)	6 (8.0%)	
Gender						
Male	600 (34.5%)	335 (39.7%)	190 (29.5%)	51 (29.1%)	24 (32.0%)	<.001^b^
Female	1138 (65.5%)	509 (60.3%)	454 (70.5%)	124 (70.9%)	51 (68.0%)	
Chronic illnesses						
No	1322 (76.1%)	621 (73.6%)	506 (78.6%)	134 (76.6%)	61 (81.3%)	.100^b^
Yes	416 (23.9%)	223 (26.4%)	138 (21.4%)	41 (23.4%)	14 (18.7%)	
Highest education level, n (%)						
Primary/secondary	328 (18.9%)	154 (18.2%)	138 (21.4%)	27 (15.4%)	9 (12.0%)	.085^b^
Tertiary	1410 (81.1%)	690 (81.8%)	506 (78.6%)	148 (84.6%)	66 (88.0%)	
Socioeconomic status, n (%)						
Low income	245 (14.1%)	95 (11.3%)	110 (17.1%)	32 (18.3%)	8 (10.7%)	<.001^b^
Lower middle-income	710 (40.9%)	303 (35.9%)	294 (45.7%)	75 (42.9%)	38 (50.7%)	
Upper-middle-income	723 (41.6%)	408 (48.3%)	227 (35.2%)	63 (36.0%)	25 (33.3%)	
High income	60 (3.5%)	38 (4.5%)	13 (2.0%)	5 (2.9%)	4 (5.3%)	

Abbreviations: SD, standard deviation; min, minimum; max, maximum.

a Kruskal-Wallis test.

b Chi square test.

### Associations Between Psychological Distress, Chronic Illnesses, and Vaccine Acceptance

**Figure 1.**
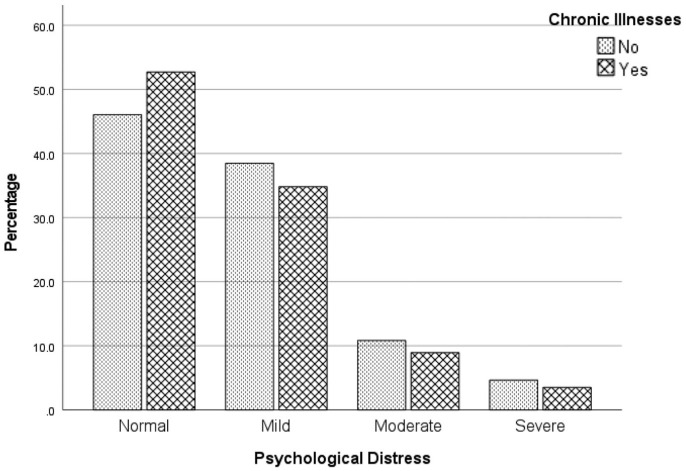
Association between psychological distress and chronic illnesses among participants who were willing to accept the COVID-19 vaccine with at least 95% effectiveness level.

Figure 1 reveals that participants with chronic illnesses who experienced a normal level of psychological distress were found to be in favor of the vaccine (53%). However, in comparison to those without chronic illnesses, vaccine acceptance was less likely for participants with a combination of chronic illnesses and an elevated psychological distress level. Specifically, the vaccine acceptance rate was reported to be 35% for those with a mild distress level, only 9% for participants with a moderate distress level, and 3% for participants experiencing a severe distress level. Overall, there was no significant association between psychological distress and chronic illnesses for those in favor of the vaccine (χ^2^[3] = 4.63, *P* = .201).

### Mediation Analysis

In Step 1 of the mediation analysis, the results showed that the relationship between psychological distress score and the willingness to accept the vaccine with at least 95% effectiveness level was significant (χ^2^[1] = 5.42, *P* = .020). Similarly, there was a significant relationship between psychological distress score and the chronic illnesses (χ^2^[1] = 9.67, *P* = .002) in step 2 of the analysis. In step 3, the relationship between the chronic illnesses and the willingness to accept the vaccine was marginally significant (χ^2^[1] = 3.65, *P* = .052). The last step of the analysis showed that the effect of the psychological distress score remained as a significant predictor for the willingness to accept the vaccine with at least 95% effectiveness level (χ^2^[1] = 4.82, *P* = .028) when chronic illnesses was also included in the regression model. Therefore, there was a partial mediation effect of chronic illnesses in the relationship between psychological distress and the willingness to accept the vaccine.

Table 2 shows that the median psychological distress score was statistically significant between participants who were willing and not willing to accept the vaccine (*U*[1] = 2.784, *P* = .005). There was a marginal significant association between chronic illnesses and the willingness to accept the vaccine (χ^2^[1] = 3.66, *P* = .052). The association of psychological distress with vaccine acceptance was further explored in different logistic regression models. After adjusting for age, gender, and socioeconomic status, elevation of psychological distress did not present any influence on vaccine acceptance (OR = 1.01, 95% CI [0.96, 1.05]). Furthermore, participants with chronic illnesses were found to be in favor of the vaccine (OR = 1.20, 95% CI [0.89, 1.61]).

**Table 2.** Association Between Psychological Distress and the Acceptance of the COVID-19 Vaccine With at Least 95% Effectiveness Level.

	Willingness to accept the vaccine with at least 95% effectiveness		*P*	Model 1^a^		Model 2^b^	
	No (n = 372)	Yes (n = 1366)		OR	95% CI	OR	95% CI
Psychological distress score							
Median (min-max)	2 (0-12)	3 (0-12)	.005^c^	1.05	1.01-1.10	1.01	0.96-1.05
Chronic conditions							
No	269 (72.3%)	1053 (77.1%)	.052^d^	1.00		1.00	
Yes	103 (27.7%)	313 (22.9%)		0.79	0.6-1.03	1.20	0.89-1.61

Abbreviations: OR, odds ratio; CI, confidence interval; min, minimum; max, maximum.

a Model 1: Logistic regression analysis without any adjustment.

b Model 2: Logistic regression analysis with adjustment for significant demographic factors such as age, gender, and socioeconomic status.

c Mann-Whitney *U* test.

d Chi-square test.

## Discussion

This study was primarily conducted to examine the mediating effect of chronic illnesses in the relationship of psychological distress and the willingness to accept the vaccine with at least 95% effectiveness level. Overall, the findings from this study showed that psychological distress has a significant influence on vaccine acceptance. Recent studies have highlighted the adverse effects of the COVID-19 pandemic on people’s mental health. Furthermore, psychological distress has been found to be positively associated with the intolerance of the uncertainty of the pandemic.^20^

The prevalence of psychological distress in this study was found to be 51.4%, which is more than two times higher than the rate reported in China^10^ (21.2%). Furthermore, the current study highlighted that about 14.4% of participants reported moderate to severe symptoms. This prevalence rate was comparable with the rate reported in Germany and Austria^21^ (19%), America^22^ (11.2%), and Saudi Arabia^23^ (14.5%), but a much lower rate was recorded in China^10^ (3.3%). The varying degrees of reported psychological distress across different countries are likely due to the demographic differences and cultural differences between the study populations.

Age was found to be negatively associated with psychological distress. A previous study showed that lower stress related to COVID-19 in daily life was reported by older adults compared with younger adults.^24^

In this study, psychological distress was negatively associated with vaccine acceptance. A possible explanation for this finding may be linked to psychological stress and vaccine-related worries. For example, anxiety symptoms in relation to the influenza vaccination was found to be correlated with pessimistic opinions of the vaccine safety.^25^ In contrast, other studies revealed that being depressed and more anxious had a slightly positive influence on the acceptance of the COVID-19 vaccine.^26^ Due to inconsistencies in the findings, it is challenging to confirm whether the reported psychological symptoms in these studies are specific to COVID-19.

Adults with underlying chronic illnesses regardless of age are at a higher risk for severe illness from the virus that causes COVID-19. For example, recent studies highlighted that there was an increased risk of in-hospital mortality among patients with multiple comorbidities. Specifically, among patients with confirmed COVID-19, about 60.8% deaths were reported for those with one to four underlying chronic illnesses, while 26.5% deaths were captured for those with five or more chronic illnesses.^27^ In addition, older adults of male gender were associated with a higher risk of COVID-19 deaths.^28^ Therefore, these adults are highly recommended for the COVID-19 vaccine.

An infodemic is defined as an overwhelming amount of information in relation to the COVID-19 pandemic available in both online and offline platforms. This platform could also present opportunities to deliberately disseminate wrong information or fake news to the public. Such misinformation can be harmful to the physical and mental health of people.^29^ Therefore, it is important for government organizations and experts to deliver compelling content about the COVID-19 vaccine in an innovative way to strengthen trust and credibility toward it among the public.

There were some limitations in this study. First, the responses captured in this online survey were done prior to the national rollout of the vaccination program in Malaysia. Second, the results from this study are likely to be biased due to the presence of recall bias and selection bias in the nature of a cross-sectional study design. On the other hand, despite about a quarter of the participants reporting having a chronic illness, this study suggested that chronic illnesses partly mediate the relationship between psychological distress and vaccine acceptance. Future studies are warranted to further ascertain the mediating effect of chronic illnesses by incorporating a more representative sample using a longitudinal study design.

## Conclusions

In conclusion, this study suggested the presence of the partial mediating effect of chronic illnesses in the relationship between psychological distress and the willingness to accept the vaccine. In order to promote the uptake of the COVID-19 vaccine, more compelling content especially for those with underlying chronic illnesses should be included in the targeted health campaigns. With the accurate information being disseminated, it is the ultimate goal of these health campaigns to reduce the psychological distress among those adults who are eligible for the vaccine and subsequently to increase their positive attitudes toward the vaccine.
